# The effect of staging of fluidic oscillation on microbubble generation in viscous liquids

**DOI:** 10.1140/epjs/s11734-025-01927-y

**Published:** 2025-09-24

**Authors:** Pratik Desai, Sarah F. R. Taylor, Johan Jaquemin, Christopher Hardacre, William B. Zimmerman

**Affiliations:** 1 Perlemax Ltd., 318 Broad Lane, Sheffield, S3 7HQ UK; 2 School of Chemical Engineering and Analytical Science, The Mill, University of Manchester, Manchester, M13 9PL UK; 3 Department of Materials Science and Nanoengineering (MSN), Mohammed VI Polytechnic University (UM6P), Lot 660-Hay Moulay Rachid, 43150 Ben Guerir, Morocco; 4 School of Chemical, Materials and Biological Engineering, University of Sheffield, Mappin Street, Sheffield, S1 3JD UK

## Abstract

Microbubbles are broadly defined as gas–liquid interfaces smaller than 1 mm and larger than 1 μm. Conventional modes of microbubble generation (ultrasound, ablative technologies, and electrolytic mechanisms) requires large amounts of energy and generate a wide distribution of microbubbles. Fluidic oscillation is an energy-efficient technique used for microbubble generation capable of generating a fairly monodisperse population of microbubbles compared to conventional bubbling. This study hypothesises that partial liquid wetting of membrane pores further benefits microbubble production. The partial liquid wetting can be introduced by utilising the ‘off’ portion of the fluidic oscillator, resulting in liquid imbibition. This liquid imbibition temporarily changes the membrane dynamics with intrapore wetting by the liquid. Therefore, using steady flow immediately after this operation would result in a similar behaviour (smaller bubble size) temporarily. This results in an interesting interplay between the two conditions—conventional bubbling (steady flow) and oscillatory flow, thereby presenting an intermediate condition with an associated reduction in bubble size when staged appropriately, i.e., for steady flow bubbling post-fluidic oscillator application. The resultant average bubble size is 25% lower than the steady flow bubbling prior to fluidic oscillation application but is dependent on physico-chemical properties: primarily viscosity and wetting angle. An intermediary condition is set based on the staging resulting in a 25–50% reduction in bubble size from steady-flow dynamics post-application of the oscillatory flow.

**Graphical abstract:**

Lubrication and hysteresis effect

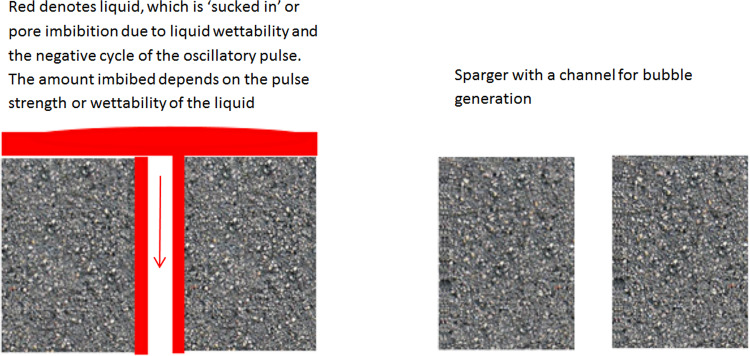

**Supplementary Information:**

The online version contains supplementary material available at 10.1140/epjs/s11734-025-01927-y.

## Introduction

To qualify as a contribution to a special edition celebrating the works of Etienne Guyon, the topic should manifestly relate to some of his important works and their findings. This paper introduces microbubbles into highly viscous ionic liquids, exploiting a hydrodynamic instability of fluidic oscillation for their generation. Hydrodynamic instabilities are a recurrent topic in the works of Guyon, with a dedicated chapter (11) in his widely read textbook [1]. The generation of microbubbles involves a complicated wetting and percolation phenomena through microporous media, typically called diffusers in the aeration and bubbling literature [2]. Percolation and wetting in porous media are common themes in the works of Guyon and co-workers, particularly due to the fractal nature of the flows [3], leading to an understanding of transient development of invasion percolation [4]. In this paper, the invasive percolation of the wetting ionic liquids is staged—a transient invasion percolation—by fluidic oscillation intervals and steady flow intervals, compared to either steady oscillation or steady flow. In their seminal work on non-locality of nonlinear problems is disordered media, Guyon et al. [5] could have laid the groundwork for this set of experiments before staging, with the direct quote:

“The medium is initially filled with a fluid **wetting** the pores. … the non-**wetting** fluid can be injected in the medium … a continuous path of non-**wetting** fluid is established across [the disordered media].”

One of the emergent features to be shown in this paper, of the staged flow, is a hysteresis or memory effect, which have been observed in domain structure transitions in liquid crystals in a highly cited work of Guyon et al. [6]. Indeed, hysteresis is mentioned three times—hysteresis effects leading to limit cycles (p. 106), the Landau formalism describing flow instabilities resulting in hysteresis or complete loss of symmetry (p.417), and hysteresis due to coexistence of gas and liquid phases—in his heralded textbook [1].

Microbubbles are broadly defined as spherical gas–liquid interfaces, sized between 1 and 999 μm. This size offers a large interfacial area, which greatly increases the surface area-to-volume ratio (SA/V) associated with them. This increased SA/V is responsible for microbubbles acting as extremely good vectors for gas–liquid transfer operations and other associated transport phenomena [7–13]. Economically feasible generation of microbubbles has been difficult to achieve. This hampers their extensive use in industry. With the advent of fluidic oscillation for microbubble generation, upscaling to industrial scale is economically feasible for some applications, e.g., wastewater aeration and flotation [14], with headway in other applications made for a feasible large-scale energy and economically efficient microbubble generation system [15–18].

**Fig. 1:**
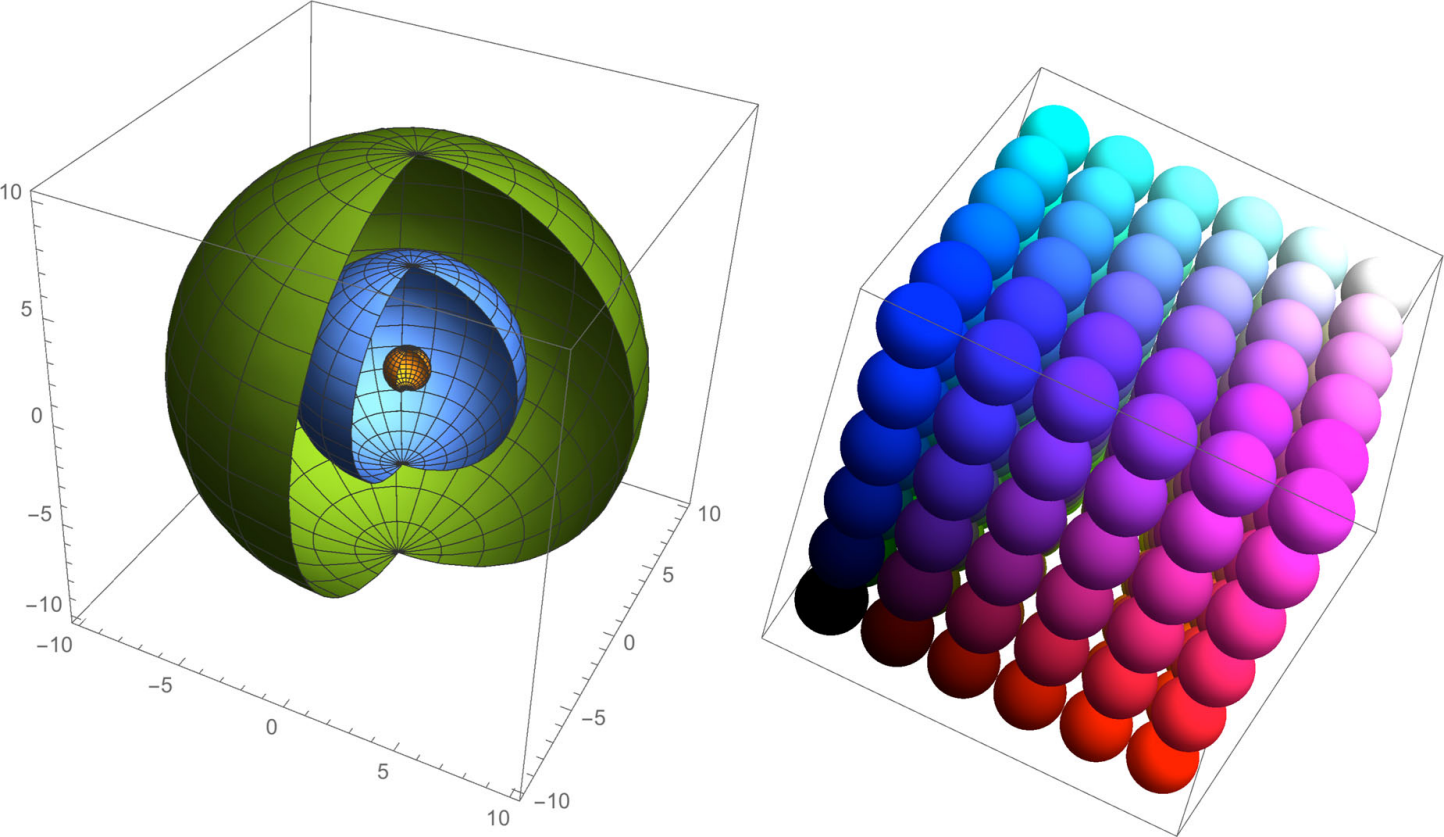
Bubble volume and surface area connotations. The figure on the right shows how bubble volume affects the surface area and the figure on the left shows how unit radii of 1, 5, and 10 have for bubble surface area and volume

Figure 1 shows the effect of the bubble surface area-to-volume ratio. To the left are three bubbles of unit radii 1, 5, and 10, respectively. The volumes and surface area can be seen therein. 1000 bubbles of unit radii 1 would be required to occupy the same volume as that of a single bubble of unit radius 10.

With three surfaces (gas inside, liquid outside, as well as the gas liquid interface) available, an extremely large residence time, increased interior convective current, ability to acquire charge on the surface using the right combination of gas and liquid, microbubbles can be extremely versatile and utilised in a variety of ways [9, 10, 16–26].

### Microbubble generation

Different ways for microbubble generation are discussed in Desai and Zimmerman [2], Zimmerman et al*.* [15], and Stride et al*.* [27]. The major cost associated with microbubble generation is their cost in terms of surface energy. Smaller the bubble, larger is the bubble surface energy required to generate it. This is governed by the Young–Laplace equation

1 $$\Delta P=\frac{2\sigma }{R}.$$

Reduction in bubble size results in a large increase in the surface area-to-volume ratio. An example is shown in Fig. 1.

The fluidic oscillator is a no-moving part, fluidic device that creates hybrid synthetic jets—oscillations with more forward flow than backflow, so that there is a net throughput. Fluidic oscillators can generate bubbles of the order of the orifice used to engender the bubbles [14, 28–33]. This Y-type bistable valve is able to switch the flow supplied to it into its two outlets exploiting the Coanda effect. The Coanda effect is the tendency of the jet to adhere to the curved surface—in this case the wall. Tesař et al*.* [30] show the most popular configuration of fluidic oscillators in use, known as the Spyropoulos loop-type oscillator. A feedback loop is attached at the control terminals of the oscillator to induce a frequency change. The incoming jet enters via the supply nozzle and is amplified at the throat via a constriction of appropriate size. Control terminals actuate the flow switching due to the emergent pressure differential (Venturi effect) between the feedback loop terminals.

This frequency is dependent on the inlet flow, the length of the control loop, and the outlet terminals which are then connected to the diffusers, placed in the liquid.

Bubble generation normally depends on the balancing of surface forces, buoyancy forces, and inertial forces. Various studies and models have been developed [33–47]. For oscillatory flow, bubble generation depends (under appropriate conditions and momentum injected) on the switching frequency of the fluidic oscillator and the amplitude of the pulse [14, 30].

Bubble formation occurs in two stages: nucleation at smaller sizes and coalescence to form larger bubbles. This happens when the bubbles formed are not monodisperse, leading to a difference in rising velocity and subsequent bubble collisions. Bubble size is not uniform. There are temporal and spatial variations throughout bubble duration in the tank. The bubble size depends on the mass and heat transfer rate [11]. Bubbles size decreases with increase in salinity or more generally the ionic strength [48], due to the reduction in surface tension, whilst the coalescence of bubbles is then suppressed [48, 49]. Multi-orifice systems, however, are much more complex than a single, submerged orifice [33].

One of the main sources of complexity is that bubbles from multi-orifice systems are not spherical. This is due to variation of pressure drop across each hole, resulting in stratification of the liquid, thus deformation of shape. However, when the pressure drop across the diffuser is very high, i.e., for porous plates with very fine holes (between 20 and 200 µm), there is uniform bubbling. There is a high probability that bubbles can adhere to the diffuser surface during expansion if a hydrophilic material is used instead of hydrophilic.

Generally, bubble formation with sieve plates is considered to have two different mechanisms. In the first mechanism, as in the case of a single orifice, bubbles are generated under low gas velocities, such that every single bubble is detached individually. With the second mechanism, bubbles are formed as a consequence of jet break-up, resulting in a fine dispersion. However, with the fluidic oscillator in place, it is hypothesised that bubble formation only follows the first mechanism. In fact, it is more dependent on the frequency and amplitude of the jet. The two-stage spherical bubble formation model is given by Luo et al*.* [48]. The mechanism of bubble generation via fluidic oscillation is provided by Tesař [50, 51].

**Fig. 2:**
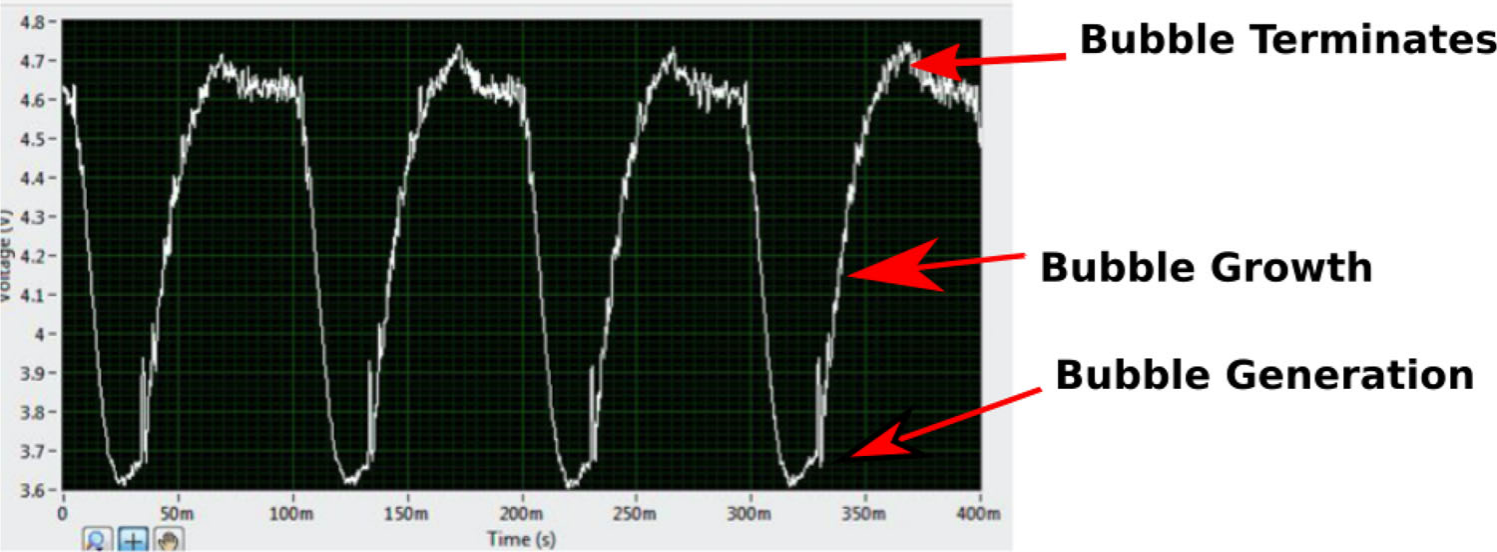
Bubble generation mediated by oscillatory flow and subsequent pinch-off for [C_2_mim][DCA]

Assumptions:

1. Bubbles are spherical in shape (since the bubbles being formed are less than the capillary length ~ 2 mm, they can be assumed to be spherical).
2. Bubbles detach from the orifice when the distance between the bubble and the nozzle reaches a critical value (when upwards force = downwards force) for conventional steady flow and during the ‘OFF’ stage for the fluidic oscillator generated bubble when the bubble pinches off during the oscillatory cycle. This can be seen in Fig. 2.
3. Bubbles are assumed to be formed in two stages, namely expansion stage and detachment stage.

With the fluidic oscillator in place, the gas is detached through the oscillatory motion rather than when the bubble expands up to a certain size. Usually, the volume of the bubble at the end of the first stage and during the second stage can be described by considering a balance of all the forces on the bubble being formed. However, the negative pulse of the fluidic oscillator works before the bubble has a chance to fully form, thus giving rise to smaller bubbles.

The overall force balance is given

2 $${F}_{B}+{F}_{M}={F}_{D}+{F}_{\sigma }+{F}_{\mathrm{Basset}}+{F}_{I,g}+{F}_{C }+{F}_{I,m}.$$

The expansion stage and detachment stage follow the same force balance equation although the expression for the same force in the two stages may be different.

*Effective buoyancy force:*

3 $${F}_{B}={V}_{B}\left({\rho }_{l}-{\rho }_{g}\right)g=\frac{\pi }{6}{d}_{b}^{3}\left({\rho }_{l}-{\rho }_{g}\right)g.$$

*Gas momentum force:*

4 $${F}_{M}=\frac{\pi }{4}{D}_{0}^{2}{\rho }_{g}{u}_{0}^{2}.$$

*Surface tension force:*

5 $${F}_{\sigma }=\pi {D}_{0}\sigma \mathrm{cos}\gamma .$$

*Liquid drag force:*

6 $${F}_{D}=\frac{1}{2}{C}_{D}{\rho }_{l}\frac{\pi }{4}{d}_{b}^{2}{u}_{b}^{2}$$

7 $${C}_{D}=24/\mathrm{Re}.$$

In the expansion stage, the rise velocity of the bubble centre is equal to the bubble expansion velocity

7 $${u}_{e}=\frac{\mathrm{d}{r}_{b}}{\mathrm{d}t}=\frac{\mathrm{d}}{\mathrm{d}t}{\left(\frac{{V}_{b}}{4\pi }\right)}^{1/3}=\frac{1}{4}{\left(\frac{3}{4\pi }\right)}^{1/3}{{V}_{b}}^{-2/3}\frac{\mathrm{d}{V}_{b}}{\mathrm{d}t}=\frac{{u}_{0}}{4}{\left(\frac{{D}_{0}}{{d}_{b}}\right)}^{2},$$

where the bubble volume is the product of the volumetric gas flow rate, *Q*, and the time, *t*

8 $${V}_{b}=Qt=\frac{\pi }{4}{D}_{0}^{2}{u}_{0}t.$$

*Bubble inertial force:*

9 $${F}_{I,g}=\frac{{\rho }_{g}{Q}^{2}{V}_{b}^{-2/3}}{12\pi {\left(\frac{3}{4\pi }\right)}^{2/3}}.$$

Bassett force is neglected in this stage.

Bubble growth and detachment is governed by the frequency of oscillation and the flow cut off induced by the oscillatory flow. Because it is a synthetic hybrid jet, the backflow phase of the oscillation results in bubble detachment comparable to the size of the orifice in question. Hanotu et al*.* [19] showed that the generation of 90 μm bubbles from 45 μm micropores is facilitated by fluidic oscillation with 1069 μm bubbles being generated from the same orifice with conventional steady flow. The oscillated outflow is a hybrid synthetic jet which underpins the major hypothesis consistent with the staging effects of the oscillator on bubble generation observed here. A hybrid synthetic jet, as described by Tesař et al*.* [28], demonstrates how bubble generation via fluidic oscillation is unique. It cannot be simply replicated using an acoustic/electronic system without compromising fidelity.

**Fig. 3:**
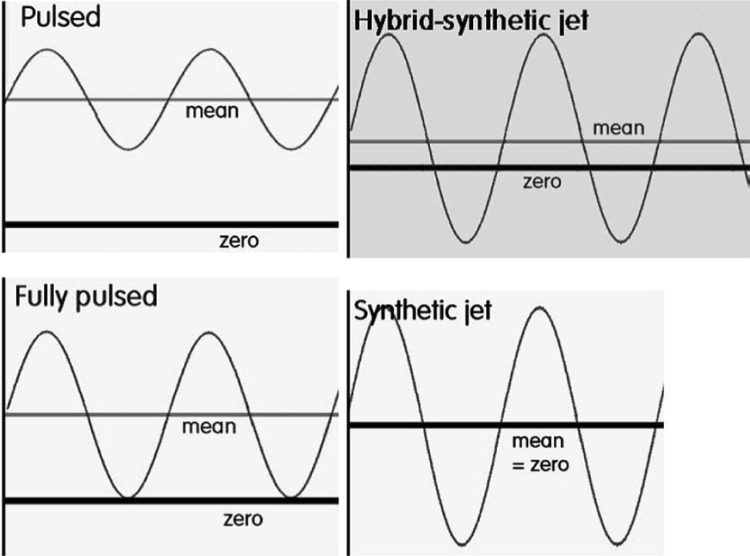
Different types of jets as described by Tesař [28, 52, 53]

Figure 3 classifies the types of jets. The fluidic oscillator generates hybrid synthetic jets which result in the net non-zero character and bubble formation uniqueness mediated by the fluidic oscillator.

This paper investigates microbubble generation via fluidic oscillation in highly viscous liquids as well as the subsequent staging effects observed that result in changes in bubble formation dynamics. It is hypothesised that the bubble formation mechanism which determines the cut off of the flow, responsible for the bubble pinch-off, results in an improvement in the conventional mechanism of bubble generation as well if staged in the right order with the caveat that it applies for viscous liquids. This is because the other factors that determine viscous forces become more dominant and responsible for bubble size [41, 54]. The negative pressure caused by the negative cycle of the oscillatory pulse results in partial liquid imbibition, which is a form of invasion percolation [4]. It is an important factor for the bubble generation mechanism, resulting in a substantial decrease in bubble size with fluidic oscillation.

The hypothesis for this paper is that the negative cycle of the oscillatory pulse results in backflow/liquid imbibition, which causes greater wetting of the orifices generating the bubbles. This forms smaller bubbles, even after the oscillator (and therefore the negative pulse cycle) has been removed, so conventional steady flow occurs, when compared to the conventional steady-flow bubble formation *before oscillator application*.

## Materials and methods

An optical visualisation setup with ionic liquids is used in conjunction with a bespoke test cell and diffuser arrangement. Bubble sizes are inferred using an optical method for three conditions: conventional steady flow, then oscillatory flow, and followed by conventional steady flow. To amplify the effect of negative backflow into the system, the chamber was sealed and maintained as a closed system. This reduce errors induced by external effects. The flow is measured at the outlet, which coupled with the bubble sizing, and provides the control required for the configuration.

### Materials

#### Chemicals used

ILs used herein are selected from those previously synthesised and tested [53]. 1-Ethyl-3-methylimidazolium dicyanamide [C_2_mim][DCA] (98%), 1-ethyl-3-methylimidazolium bis(trifluoromethylsulfonyl)imide [C_2_mim][NTf_2_] (≥ 97%), and 1-butyl-3-methylimidazolium bis(trifluoromethylsulfonyl)imide [C_4_mim][NTf_2_] (≥ 98%) are used as received from Merck. 1-Ethyl-3-methylimidazolium ethylsulfate ([C_2_mim][EtSO_4_]) is synthesised by dissolving diethylsulfate (Sigma-Aldrich, 98%, 154.2 g, 1 mol) in ice cold toluene (Sigma-Aldrich, ≥ 99.5%, 100 cm^3^) and adding this solution dropwise to 1-methylimidazole (Sigma-Aldrich, 99%, 82.1 g, 1 mol) dissolved in water (500 cm^3^) in an ice bath under a nitrogen atmosphere. This solution is stirred overnight. The organic solvent is then removed and the former IL is then sequentially washed with toluene (100 cm^3^) and dried in vacuo five times. 1-Butyl-3-methylimidazolium trifluoroacetate [C_4_mim][TFA] is synthesised from trifluoroacetic acid (Sigma-Aldrich, 99%, 114.0 g, 1 mol) added dropwise to 1-butyl-3-methylimidazioum chloride (174.7 g, 1 mol) dissolved in Milli-Q ultra-pure water (500 cm^3^) in an ice bath and allowed to stir overnight. The solvent is then removed using a rotary evaporator to obtain the IL. All ILs are dried in vacuo (< 10^–2^ mbar @ 40 °C) for a minimum of 48 h and maintained under a flow of dry N_2_ overnight before microbubble experiments are conducted. After drying, the water content of the ILs is measured using a Metrohm 787 KF Titrino Karl Fischer as < 0.1 wt% for all ILs. The purity of the synthesized ILs is analysed using ^1^H NMR using a Bruker 300 MHz Ultra shield Plus NMR spectrometer. The results were consistent with the literature reports [56, 57].

Air is supplied from a pressurised compressor at 8 bar (g). This stream is then regulated by a pressure regulator down to the bubbling pressure required for the target volumetric flowrate.

Two setups have to be used for this with one connection maintained with a back flow. The systemic pressure is controlled using a pressure regulator and the flow is controlled using a mass flow controller. The oscillator is then connected to the test cell setup. There is a limited amount of flow allowed through the system to maintain pressure levels below that of a pressurised vessel. The pressure is maintained, so that the system is as close an approximation to closed as reasonably possible. Flow matching is performed using a mass flow controller (Bronkhorst—miniCoriFlow) placed at the outlet.

The light source and camera are placed opposite each other with the rig in the centre to obtain maximum contrast and collect the best images possible. Conventional steady flow is used first to generate bubbles at the fixed flow rate.

Maintaining similar conditions, the oscillator is then introduced to the system and the bubbles are sized again. This is then replicated with conventional steady flow. The former defines the staging effect we are investigating, whilst the latter is the control for comparison purposes of the influence of the active principle of staging oscillation before steady flow for bubble generation.

### Bubble generating system

This cell has been specifically designed for generating bubbles using small samples (50–100 cm^3^) and with glass windows to size the bubbles generated using a high-speed camera. The test cell has been designed for low flow systems with a maximum liquid volume of 100cm^3^ and flowrates in millilitres per minute (mlpm), and housing a ceramic multiporous diffuser (HP Technical Ceramics, Sheffield UK; fused sintered alumina, 20 μm average pore size, 2 off—50 × 25mm^2^ area).

**Fig. 4:**
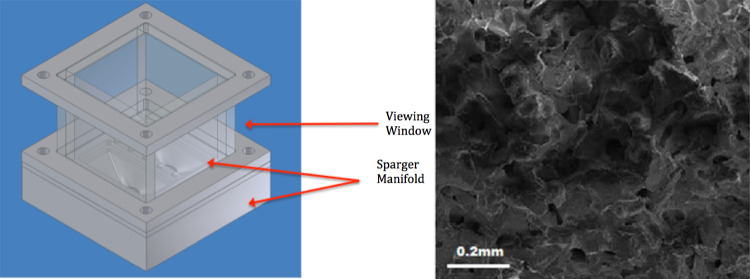
Test cell. With a viewing window and SEM of mesoporous diffuser used in this study

Figure 4 shows the test cell, and an SEM image of the ceramic diffuser used. The visualisation window (quartz windows) allows bubble size determination by optical imaging. The diffuser can be changed to collate the best size distribution possible. Scanning electron microscopy (SEM) is carried out on a JEOL JSM 6300 SEM with an Agar MB7240 gold sputter coater. This diffuser has a thickness of 5 mm and the pressure required to allow bubbling in an aqueous system is 40 mbar(g) at 298 K and 101.325 kPa.

#### Pneumatic setup

**Fig. 5:**
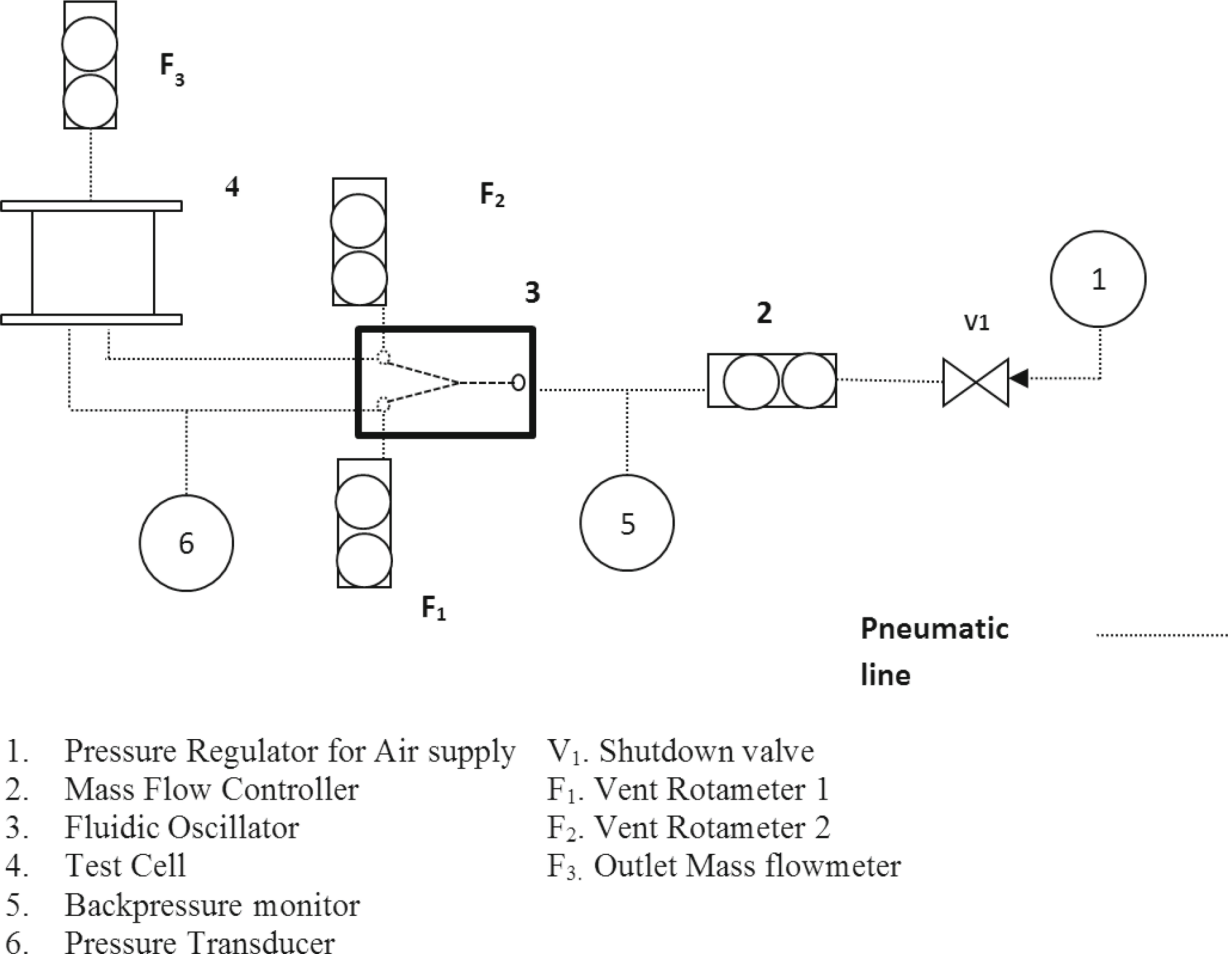
Schematic of the setup

Gas enters the system via the pressure regulator which sends out a controlled, well-regulated gas supply into a mass flow controller. The mass flow controller feeds in the appropriate amount of air to flow into the fluidic oscillator to initialise oscillation. Pressure sensors and backpressure monitors indicate the pressure and energetics of the system (Fig. 5).

As discussed previously, the test cell has been designed to work with low volumes of liquids and therefore low flow (cm^3^ per minute, i.e., millilitres per minute, rather than litres per minute required to initialise the oscillations) enters the test cell, whereas a much larger volume of gas enters the oscillator. The gas enters via the pressure regulator at a fixed known pressure. A ball valve acts as an emergency safety valve, *V*_1_. Rotameter labelled 2 is used to control the flow appropriate to the FO to initiate oscillation. *F*_1_ and *F*_2_ act as vents and are metered valves (rotameters). Pressure transducer labelled as 4 is used to measure the pressure and frequency of oscillation. 5 is the test cell and described in Fig. 4. The backpressure monitor and the pressure transducers allow pressure drop measurement across the fluidic oscillator as well as characterising the amplitude and frequency of the fluidic oscillator. The vent rotameters guide the actuation of the oscillator for sending the appropriate rate of gas into the test cell. The flow metre at the end of the test cell helps to determine the flow delivered into it, as vent rotameters cannot be used to measure oscillatory flow. This is so that oscillated air of an appropriate frequency, amplitude, and flow rate can be delivered to the test cell. The pressure transducer located at the outlet of the oscillator measures the amplitude and frequency of the oscillated air pulse which has been used to calculate flow through the oscillator, upstream of the diffuser. The flows for the system are monitored and adjusted using a mass flow metre (Bronkhorst model—Mini-CoriFlow) placed at the outlet of the system. The liquid layer in the system provides sufficient resistance which mitigates against any additional damping effects of the oscillatory flow by the mass flow controller.

### Frequency measurement

The frequency of the oscillator is determined using a bespoke code written in LabView. This could also be used to determine the magnitude of the pulse strength on the oscillatory wave. The code consisted of a fast Fourier transform (FFT) power spectrum for the raw data obtained from the pressure transducer at 128 kilo-samples per second. The FFT is a signal processing technique which, when respecting the Nyquist criteria and with sufficient sampling rate, results in a sampling averaged frequency of a wave power spectrum yielding in a peak formed for the various systems in use. The frequency of oscillation is needed to infer the associated the amplitude of the wave jet emanating from the oscillator outlet into the diffuser.

### Imaging setup

The bubble sizing and imaging setup is described in Brittle et al. [7], and Wesley et al*.* [58, 62]. The test cell is a specially designed bubble generator and described in Fig. 4. It is placed in the centre with the camera and diffused light sourced placed opposite each other to obtain maximal contrast.

The high-speed camera (Pixelink PL742 camera) is placed antipodal to the diffused light source (Thorlabs LIU004-intensity—1700 W/cm^2^ and 450 nm—emission peak) with the test cell placed in the middle. The bright LED light source is diffused into a more uniform light using a white plastic translucent optical diffuser layer, placed before the test cell with the visualisation windows where the bubbles are imaged with the camera. Bubble sizes are determined by a bespoke code developed in LabView. Bubble size analysis is performed and histograms are generated with the code from the images captured. Mean, mode, and median bubble sizes were calculated as described below.

Mean stands for the average bubble size and is calculated using

10 $$D\left[\mathrm{1,0}\right]=\overline{D }= \frac{\sum_{i=1}^{n}{D}_{i}}{n},$$

where *D*[1,0] is the mean, *D*_*i*_ is the diameter of the bubble *i*, and *n* is the number of bubbles.

Mode stands for the maximum bubbles of the same size appear in the histogram or the bubble size that appears most times in the histogram.

Median stands for the middle value of bubble size in the histogram or the bubble size which lies right in the centre of the histogram. This value is also commonly denoted as *D*_50_ in particle sizing [58, 59].

The low air flow into the system also facilitated the use of the optical bubble sizing technique as a single plane can be observed and imaged, which minimises the magnitude of the error in the measurement technique. Three repeats were taken.

### Contact angle measurement

The contact angle measurements are made using an Attension pendant drop tensiometer for the IL. The methodology is described in Brittle et al*.* [60]. The ILs are kept in a dry environment and heated to 60 °C for 24 h to remove residual moisture whilst maintaining vacuum desiccation in the presence on CaCl_2_. The Attension pendant drop tensiometer is able to take precise measurements of the contact angle using a visualisation setup similar to the bubble sizing visualisation setup including a monochromatic light source and an adjustable sample using software able to recognise the drop and measure its contact angle. The ILs are pipetted onto the cleaned substrate stage, adjustable in three dimensions using micrometre screws. The droplet is then centred and an image taken for analysis using the software. An averaged contact angle is calculated from the recorded imaging taking the left and right angles into consideration (within 5% of each other or symmetry).

### Bubble size analyses

Two factors are readily computable for the bubble size analysis- average bubble size in terms of number of bubbles and average bubble size in terms of void fraction contribution (volume contribution) of the bubble. Depending on the application, bubble sizing is usually reported using either of these two factors. Since number contributions are more relevant for this paper and generally more widely used, all discussions related to bubble sizes except when specifically stated are in terms of number contributions.

Table 1 provides an exemplar for a case wherein there are 3 classes of bubbles—Class A with a size of 1 μm and 600 in number, Class B with size of 100 μm and 200 in number, and Class C of size 500 μm and 200 in number. This leads to a total of 1000 bubbles. The surface area of the bubbles is measured and so is the volume. The bubble size can be computed by either using average bubble size in terms of numbers, i.e., weighted bubble size divided by total numbers, or in terms of average bubble size in terms of volume contribution, i.e., weighted bubble volume divided by total bubble volume.

**Table 1:** Exemplar

S. no.	Bubble	Size	Number	Volume of individual bubble	Total volume contribution	Surface area	Total surface area	Surface area/volume
1	A	1	600	5.24E-01	3.14E + 02	3.14E + 00	1.88E + 03	6.00E + 00
2	B	100	200	5.24E + 05	1.05E + 08	3.14E + 04	6.28E + 06	6.00E-02
3	C	500	200	6.54E + 07	1.31E + 10	7.85E + 05	1.57E + 08	1.20E-02
			1000		1.32E + 10			
*N*_Av_	120.6 µm	*N*_VC_	200 µm					

Average bubble size in terms of number

11 $${N}_{\mathrm{av}}= \sum_{i=1}^{n}\frac{{n}_{i}{x}_{i}}{n},$$

where* n* is the total number of bubbles and *n*_*i*_ is bubble contribution for each bubble of size *x*_*i*_*.*

The average bubble size in terms of volume contribution is

12 $${N}_{\mathrm{vc}}= \sum_{i=1}^{n}\frac{{n}_{i}{V}_{i}}{nV}$$

with

13 $${V}_{i}=\frac{4}{3}\pi {{x}_{i}}^{3},$$

where *n* is the total number of bubbles and *n*_*i*_ is the bubble contribution for volume of each bubble of size *x*_*i*_ represented by *V*_*i*_*.*

This also means that 1 million 1 µm bubbles occupy the same volume as a single 100 µm bubble. This brings about a massive disparity in bubble size in terms of volume contribution. However, the volume contribution is a useful factor for estimating any transport phenomena. It is more appropriate than averaging over number contribution.

Generally speaking, size distributions collated from membranes are narrow and the difference in the two averages is lower. A large difference in bubble sizes is observed for a highly dispersed distribution and it is beneficial to the system to have a narrower size distribution. This exemplar demonstrates how these two values need not be the same and their dispersity results in the width of the bubble size distribution. Work by Allen [53] and Merkus [60] explain the nuances associated with particle sizing and statistical calculations performed for them in detail.

## Results

Bubble size distributions are measured for various conditions with the different ionic liquids. Fluidic oscillator application resulted in a bubble size reduction for all the cases. A general result is provided for the gamut of ILs studied here, but detailed discussions are made for an exemplar.

The graphs show the bubble size variations for the three conditions and follow the staging order.

*Steady flow* is the first condition, followed by the *fluidic oscillator flow* condition and then followed by *steady-flow post-fluidic oscillation* condition.

The bubble size variations and the effect of the oscillator can be observed and the differences are within experimental errors (less than 5%). There is not only a substantial decrease in bubble size observed due to the introduction of the fluidic oscillator, but there is also a concomitant increase in bubble throughput as well as a uniform bubble generation across the membrane.

**Fig. 6:**
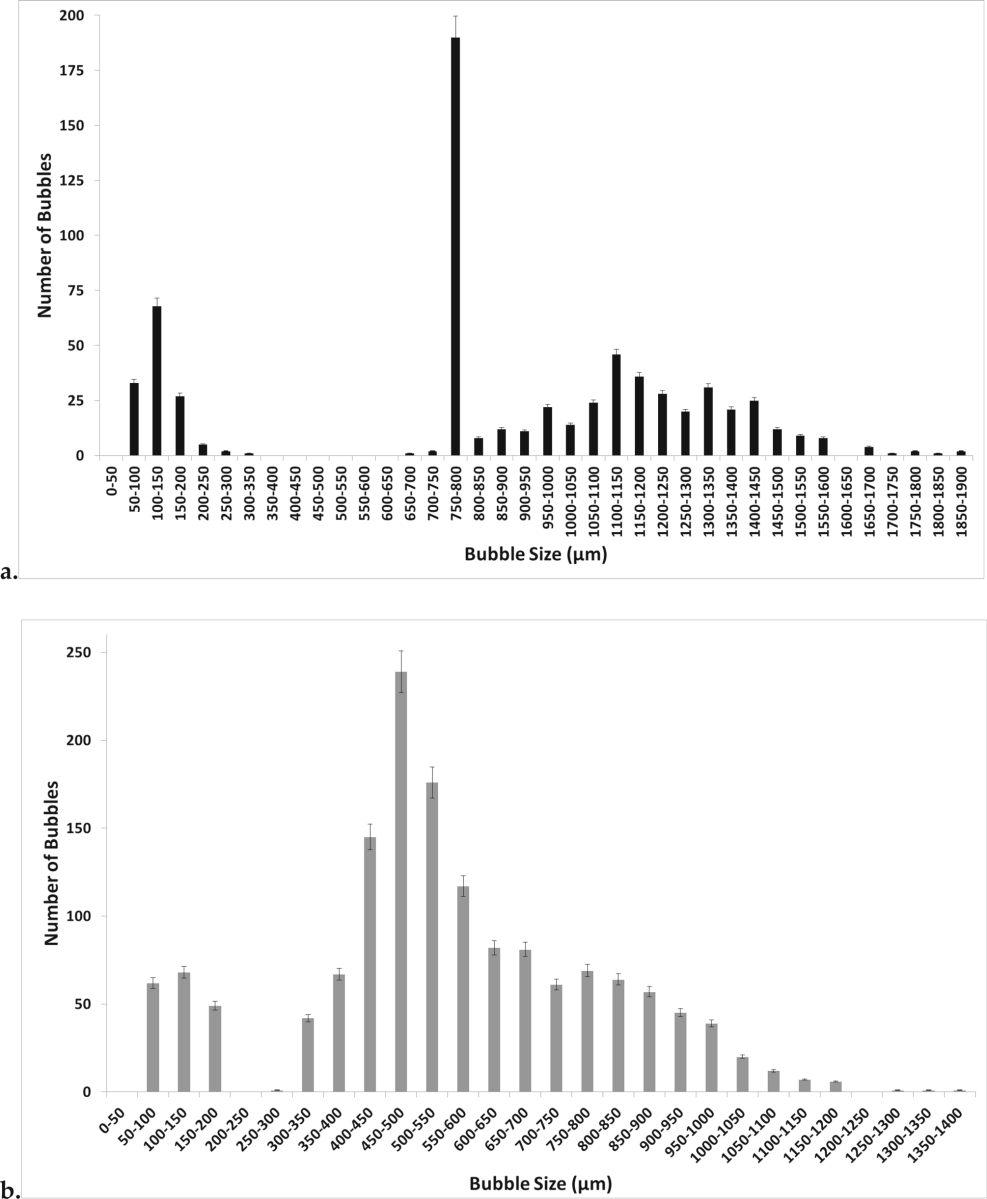
Bubble size distribution for (**a**) steady flow and (**b**) for fluidic oscillator generated bubbles in [C_4_mim][TFA]

Figure 6 demonstrates the difference in the bubble size distributions at steady flow and oscillatory flow for [C_4_mim][TFA]. The number of bubbles (bubble throughput) is increased significantly for oscillatory flow. The bubble size distribution is wide, and since larger bubbles are formed, fewer bubbles are formed for the same throughput for conventional steady flow. The bubble size distribution is narrower and far more numerous bubbles are formed and the size distribution can be seen to shift to the left for fluidic oscillator-mediated microbubbles in [C_4_mim][TFA].

**Fig. 7:**
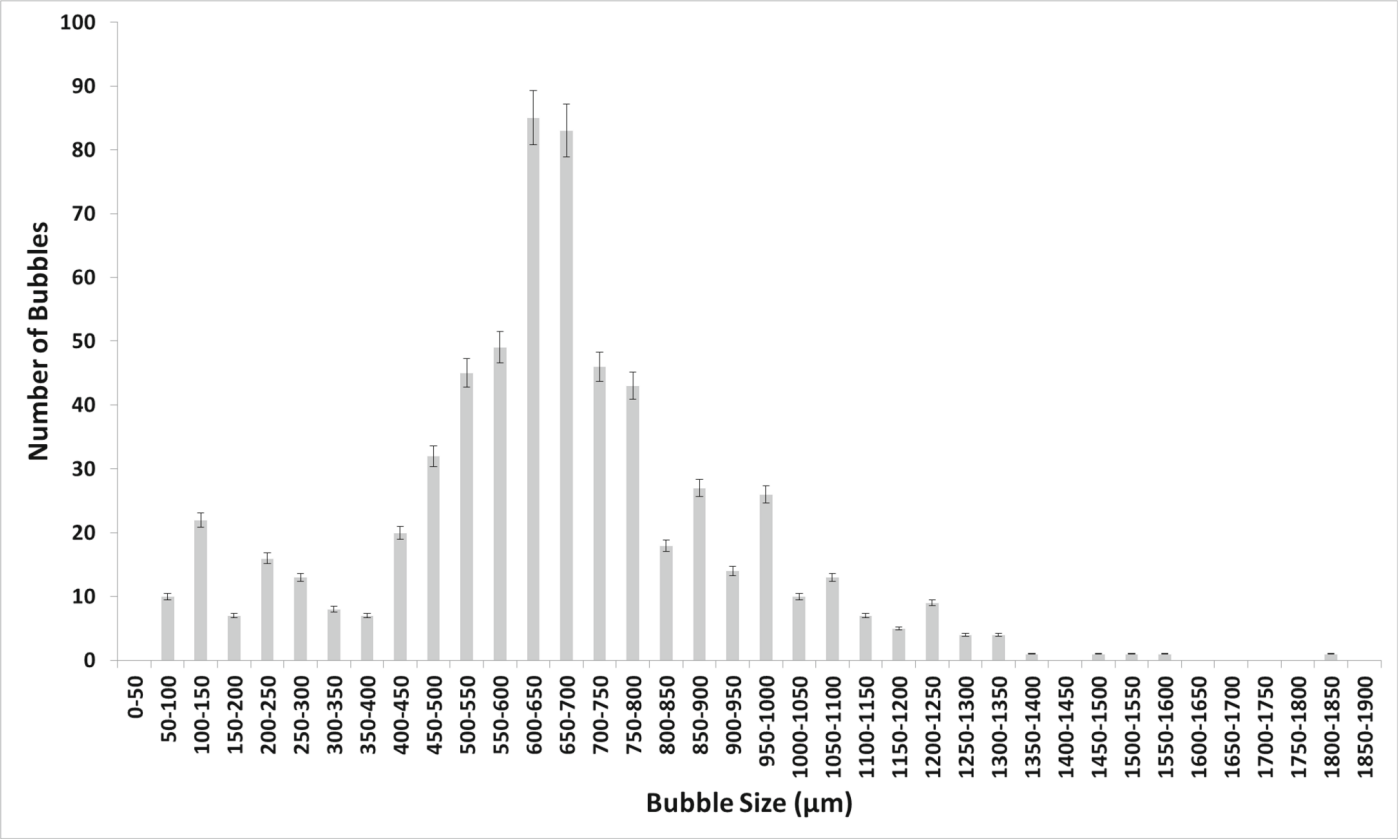
Bubble size distribution for conventional steady-flow post-FO in [C_4_mim][TFA]

Figure 7 shows the bubble size distribution for conventional steady-flow post-fluidic oscillator implementation in [C_4_mim][TFA].This shows a 'morphing' of the two bubble size distributions. The bubble size distributions are merged and there is a slight shift to the left compared to the steady flow originally. The smaller bubbles seem to be generated in the size range for when the fluidic oscillator was applied and also produced in the size range where only steady flow had generated the bubbles. This looks like an amalgamate of the two conditions or a resonant type condition where it is neither steady-flow size distribution nor fluidic oscillator flow size distribution but exhibits properties of both the size distributions.

**Fig. 8:**
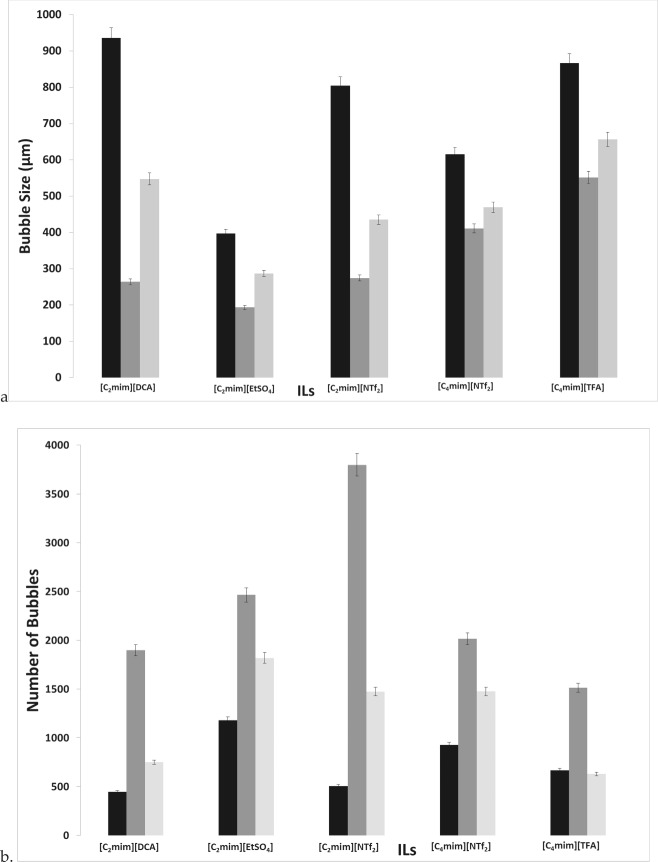
Bubble size distribution (**a**) and throughput (**b**) for the various IL exhibiting the staging effect observed —steady flow, —fluidic oscillator, and —steady-flow post-fluidic oscillator implementation

Figure 8 shows the average bubble size and throughput for the various ILs.

The bubble size decreases, and correspondingly throughput increases, with fluidic oscillator implementation compared to steady flow. The corresponding bubble size and throughput agrees with the hypothesis for steady-flow post-fluidic oscillator implementation. The steady-flow post-fluidic oscillator condition displays a mid-range value of the oscillator and steady-flow condition and retains this for a short period of time (≈ 1600–1800s). Once the system is flushed/cleaned, this feature is not retained. The throughput is seen to increase substantially upon fluidic oscillation. The bubble size has reduced by 50% for most cases with [C_2_mim][DCA] showing an approximate 3.7-fold reduction in bubble size (940 µm for steady-flow down to 260 µm for oscillatory flow) and fourfold increase in throughput (from 450 for steady flow up to 1900 for oscillatory flow). Upon subsequent implementation of steady-flow post-oscillation for [C_2_mim][DCA], there is a combination of oscillatory flow and steady flow behaviour observed, which results in a 50% reduction in bubble size (now 547 µm) compared to steady flow or 50% increase in bubble size compared to oscillatory flow and likewise for throughput (now 750). This type of behaviour is observed for all the ILs tested. The interesting behaviour to note is for [C_2_mim][NTf_2_] where the staging effect observed (steady-flow post-FO) is closer in size to the steady flow than the FO and can be seen as a direct consequence in the throughput chart. Viscosity and other physico-chemical parameters will be considered in the discussions. The important aspects can be seen in the individual bubble size distributions for the three conditions.

## Discussion

**Fig. 9:**
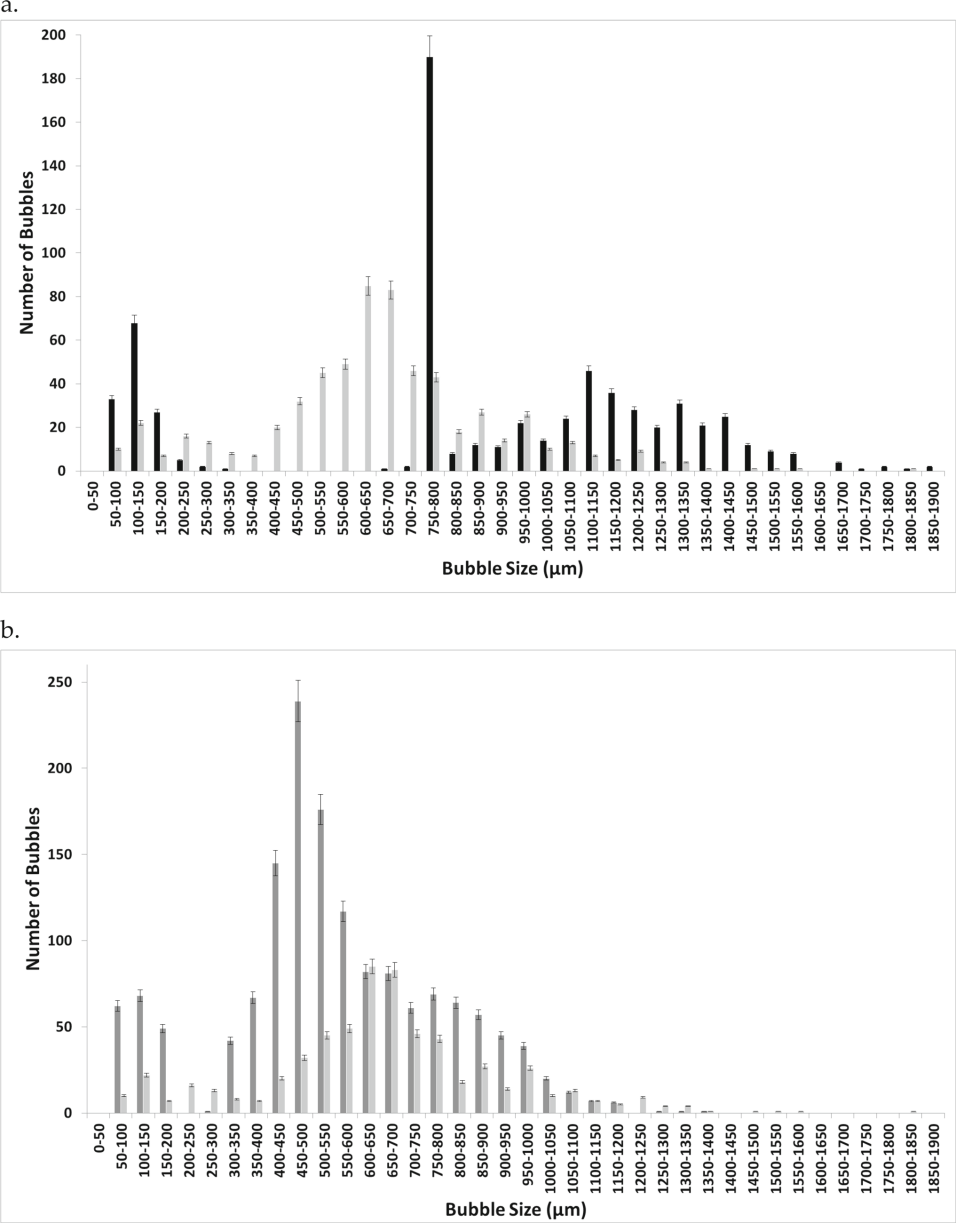
Bubble size distributions for [C_4_mim][TFA] at steady flow pre- and post-FO —steady flow, —fluidic oscillator, and —steady-flow post-fluidic oscillator implementation

**Fig. 10:**
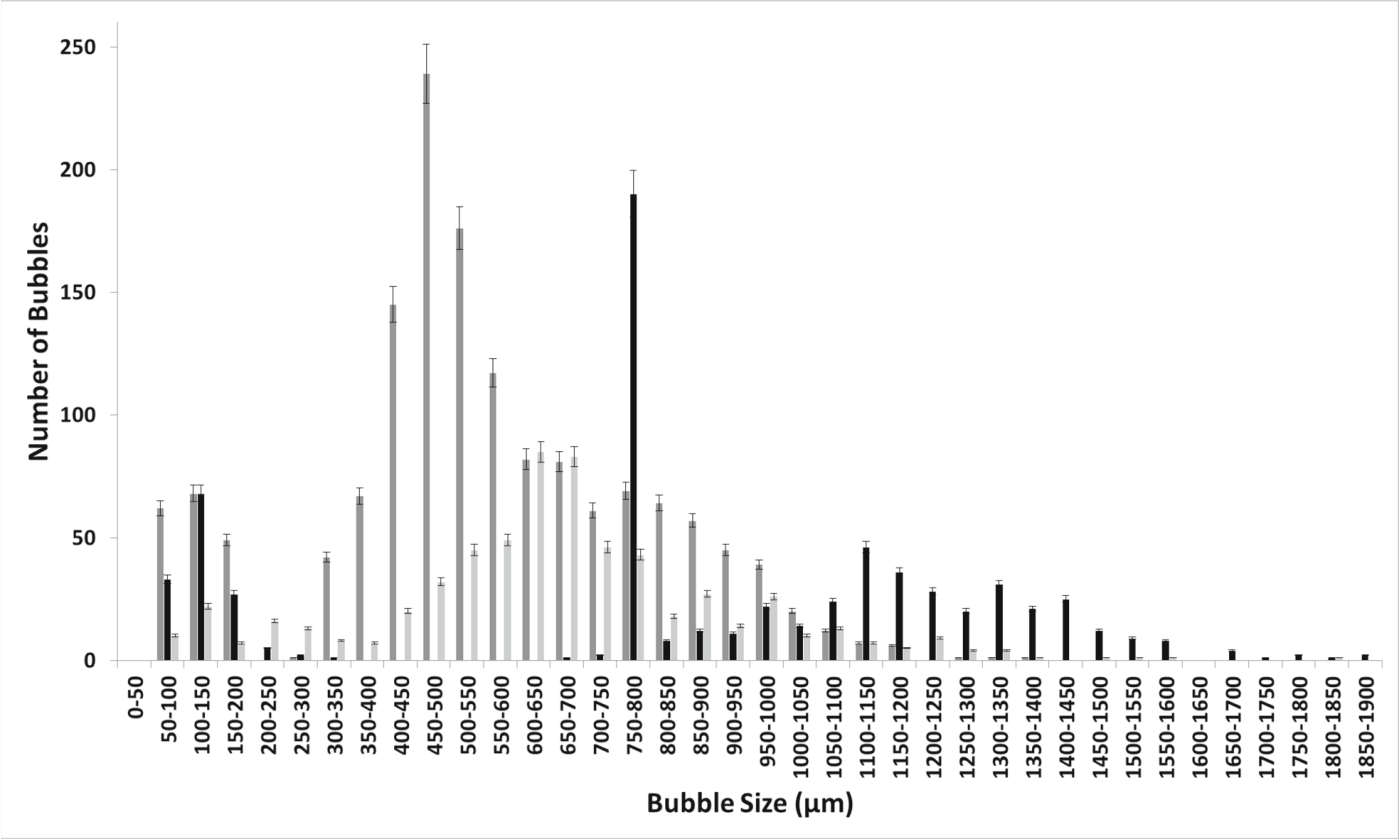
The juxtaposition of the bubble size distributions at the three conditions for [C_4_mim][TFA] —steady flow, —fluidic oscillator, and —steady-flow post-fluidic oscillator implementation

Figures 9 and 10 show the bubble size distribution for an exemplar IL [C_4_mim][TFA] being superimposed. This shows a much clearer picture of the bubble formation dynamics. The bubble size distributions show how the steady flow formation post-fluidic oscillator implementation is a mixture of fluidic oscillator-induced bubble size distribution and steady-flow induced bubble size distribution. The fluidic oscillator-induced bubble size distribution is substantially better than steady-flow-induced bubble size distribution. There is a significant reduction in the bubble size for steady-flow post-oscillator implementation due to the morphed behaviour observed in Fig. 10. However, the pseudo-oscillator-induced behaviour can be detected from this bubble size distribution. The pore activation is evident, consistent with the bubble throughput increase accompanying the size reduction.

Figure 9 shows how the bubble size distributions exhibit a high level of similarity. The smaller bubbles for post-FO steady flow resemble FO bubbles. The figure here shows the morphed characteristics of FO and steady-flow post-FO.

Figure 10 shows that the FO application results in a substantially higher number of bubbles formed as compared to both the steady-flow conditions. Similarities and the morphing of the bubble size distributions for steady-flow post-FO to a condition between FO and steady flow is observed. The pseudo-condition retains properties of both fluidic oscillator and steady-flow conditions. This results in an average bubble size smaller than the steady-flow condition but larger than the oscillator-mediated condition.

**Fig. 11:**
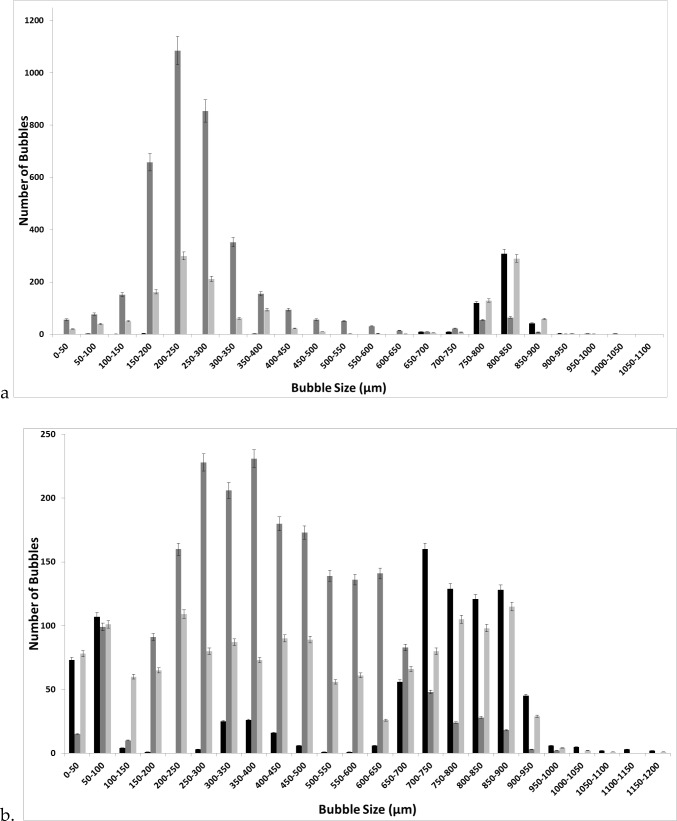
FO application for [C_2_mim][NTf_2_] (**a**) and [C_4_mim][NTf_2_] (**b**)——steady flow, —fluidic oscillator, and —steady-flow post-fluidic oscillator implementation

Figure 11 shows the difference between the bubble size distributions obtained by changing the moieties. The change in the moieties—[C_2_mim] and [C_4_mim]—changes the bubble size distribution as well as the impact of steady-flow post-FO implementation. The resulting distributions are very different. The diffused structure due to the reduced polarisation is observed for [C_4_mim] over [C_2_mim]. This leads to a broader bubble size distribution and a more diffused structure. This also reduces the difference between the FO and steady flow as well as the staging effect.

The FO application results in a substantially higher number of bubbles formed relative to both the steady-flow conditions.

**Fig. 12:**
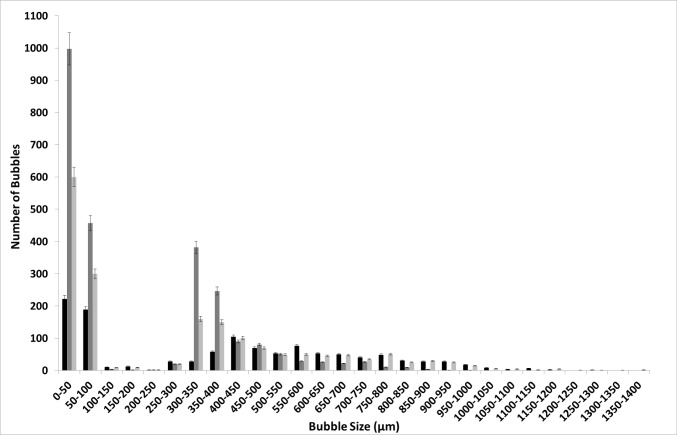
Bubble size distribution at the three conditions for [C_2_mim][EtSO_4_] —steady flow, —fluidic oscillator, and —steady-flow post-fluidic oscillator implementation

[C_2_mim][EtSO_4_] shows a distinctly similar approach due to the high polarisation between the moieties which then results in a similar bubble size distribution as for all three cases but just with an increased throughput due to the pore activation and residual FO effects for staging (Fig. 12). FO application results in a substantially higher number of bubbles formed relative to both the steady-flow conditions.

This ensures that the bubble size distribution is the same shape for all three cases but with slightly larger bubbles (at the tail end) for the steady flow and staging effect consistent with volume conservation. Moreover, the smaller bubble throughput is increased for the FO implementation case over fivefold—1000 for FO, 200 for steady flow and 600 for steady flow post-FO (staging effect). Table 2 collates information on bubble throughput and sizes for these three conditions.

**Table 2:** Table summarising bubble sizes, bubble flux, and properties of the ionic liquids used at various conditions

Ionic liquid	Conditions	Bubble sizes (µm)	Number of bubbles	Viscosity (N s/m^2^)	Contact angle (*θ*, °)
[C_2_mim][DCA]	Average bubble size (SF)	936	445		
	Average bubble size (FO)	264	1897	0.01594	43.7
	Average bubble size (SF post-FO)	547	749		
[C_2_mim][ETSO_4_]	Average bubble size (SF)	397	1179		
	Average bubble size (FO)	193	2465	0.1204	53.5
	Average bubble size (SF post-FO)	287	1819		
[C_2_mim][NTf_2_]	Average bubble size (SF)	804	506		
	Average bubble size (FO)	274	3798	0.03249	56.4
	Average bubble size (SF post-FO)	435	1474		
[C_4_mim][NTf_2_]	Average bubble size (SF)	615	926		
	Average bubble size (FO)	411	2015	0.052	36.7
	Average bubble size (SF post-FO)	469	1476		
[C_4_mim][TFA]	Average bubble size (SF)	866	666		
	Average bubble size (FO)	551	1512	0.053	35.3
	Average bubble size (SF post-FO)	657	628		

**Fig. 13:**
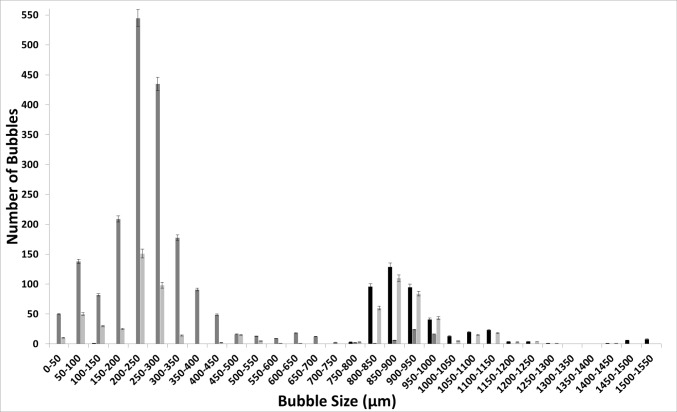
FO application results in a substantially higher number of bubbles formed relative to both the steady flow conditions for [C_2_mim][DCA] —steady flow, —fluidic oscillator, and —steady-flow post-fluidic oscillator implementation

The [C_2_mim][DCA] ionic liquid has distinctive emergent physical properties of the two-phase system (Fig. 13). The bubble size distribution is fairly distinct between the FO and steady flow and the staging effect observed is disparate. Once again, the oscillator throughput is high (≈ 6000). The steady flow bubbles are larger with the steady flow post-FO, displaying, once again, similarities to both systems as a hybrid bubble size distribution.

The size difference thus observed holds so for all the different systems explored. These systems only return to normal performance after bubbling it dry or removing the liquid and drying out the system entirely.

**Fig. 14:**
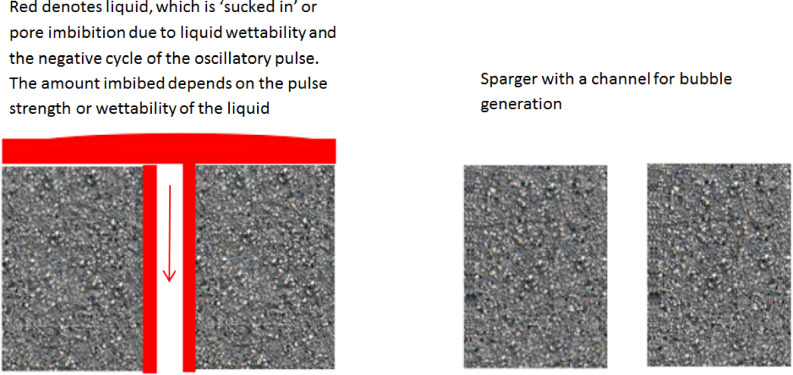
Lubrication effect. The grey area is the diffuser with the blank space (right) representing the pore channel with the shaded area representing the liquid weep into the orifice thereby providing lubrication like effect (left)

The bubble size variations observed here support the hypothesis about the staging effect of the oscillator. That backflow is introduced into the system due to the negative cycle of the synthetic hybrid jet emerging from the oscillator has been explicitly demonstrated by Tesař [28, 30, 50, 51, 61]. However, the backflow, driving invasion percolation [4], results in pore wetting and subsequent pore activation for the membrane used in conjunction with the oscillator (Fig. 14). This results in an increased wetting of the membrane by the liquid of interest. This increased wetting not only results in a reduction in bubble size, but also an increase in throughput via the pore activation.

We propose that wetting of the liquid across the membrane and into the orifice (liquid weeping) causes the decrease in bubble size [60].

Figure 16 illustrates the lubrication effect and the associated liquid weeping. The latter is determined by the liquid wetting across the surface of the membrane. However, to satisfy the hypothesis, liquid viscosity also ought to play an important role. Viscosity can be seen to be acting in opposition to the inertia of the gas that promotes bubble formation. Therefore, coupled with the liquid height above the membrane, it is the ‘resistance’ to bubble formation. With oscillatory flow, although the low contact angle, i.e., liquid wetting, is appropriate at the start for bubble formation, it is the liquid viscosity that is more important for the staging effect to take place effectively, with wetting properties constant.

Figure 2 shows the bubble formation under oscillatory flow for an exemplar liquid. The negative pulse is important for the bubble formation and for causing this staging effect.

For *τ*, being the time constant,

At *τ*_0_ − *τ*_½_, i.e., positive half cycle or ‘ON’ condition, augmenting the buoyancy forces, the bubble detaches if the amplitude matches the detachment force required and the forces are balanced.

*τ*_1/2_ − *τ*_1,_ i.e., negative half cycle or ‘OFF’ condition with a negative force (back flow) opposed to the buoyancy force. This condition induces the liquid imbibition into the porous channels. Consequently, there is an uniformly distributed liquid 'lubrication' for the pores which then homogenises the flow through, by equalising the force across the membrane due to the liquid. The next positive cycle, smaller bubbles are formed. Once the liquid wets the pores, the lubrication effect ensures that there is complete wetting observed in the system. This lubrication effect results in a tremendous narrowing of the orifice, therefore, resulting in a temporary reduction in bubble size. This could have several implications on the bubbling process. The FO generates smaller bubbles at the expense of backpressure, i.e., the pressure drop across the fluidic oscillator required to initiate the oscillation. The backpressure is extremely low − 0.2 bar. However, if some performance loss can be traded off at the expense of operating costs (higher throughput), then pulsed use of the oscillator might reduce the operating costs of the process whilst maintaining required bubble size.

When the oscillator is removed, and steady flow is applied, the behaviour observed is similar to a mixture of the steady flow and the oscillatory flow. The ‘memory’ retained is temporary but also only relative to the viscosity of the liquid and is between 1600 and 1800 s. For an IL with a lower viscosity, there is an effect of the staging on the system and the morphed character observed has a smaller bubble size. The staging observed has smaller bubble sizes and retains the characteristics of the steady flow and oscillatory flow. The higher the viscosity, the greater is the bubble size, for all conditions. However, the difference in size is minimised. One can think of this as it is the inertia of the injected jet that ensures bubble pinch-off. The oscillator results in a negative pulse which is able to weep and coat the orifices with the negative or ‘OFF’ pulse [28, 50].

The oscillatory pulse results in a hybrid synthetic jet which is an amplification as seen in the Fig. 3. If *x* is the impinging jet, then the amplification is *x* + d*x* for one leg, and *x* − d*x* for the other leg, where d*x* is the amplification of the flow by the oscillator. The d*x* term determines the level of liquid imbibition aside from the wettability and the viscosity of the liquid. The d*x* is dependent on the *x*, and amplitude of the negative pulse.

This results in a scenario where the wetting is increased substantially, and the bubble-forming surface goes from a certain amount of wetting in the ON phase, to almost complete wetting in the OFF phase. Complete wetting then results in a different regime for bubble formation as observed by Byakova et al*.* [36, 62]. The bubbles so formed will be smaller than the ones observed for when there is incomplete liquid wetting and dependent on the properties of the liquid, i.e., conventional bubbling.

This is most prominently seen in liquids with a higher viscosity as averse to liquids with a viscosity similar to water or lower. Other forces tend to dominate with low viscosity, and the question of pore activation does not arise.

The effect of staging continues as long as bubbling continues. Once the liquid is removed, the lubrication effect no longer occurs. The operation reverts to simple steady flow behaviour.

The backpressure is defined as the combined pressure drop across the oscillator, diffuser, and the height of liquid above the membrane. This also includes the bubbling pressure.

**Fig. 15:**
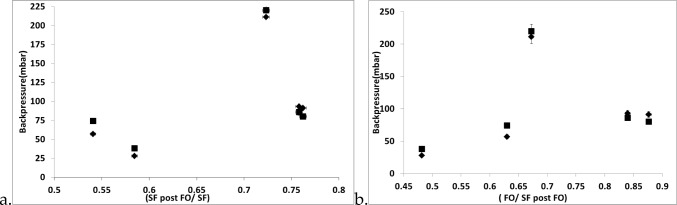
Backpressure (bubbling pressure and pressure on the system) for steady-flow post-fluidic oscillation/steady flow [left (**a**)] and fluidic oscillation/steady-flow post-fluidic oscillation [right (**b**)] for various ILs at the three conditions—actual backpressure observed, —theoretical backpressure

The figures seen in the results section demonstrate the level of changes observed in the system when the FO is introduced. For differences in bubble size with respect to physical properties, they have been dealt with in the Taylor et al*.*’s [55] study for FO and steady flow. Interestingly, the staging effect is observed here and conforms closely with the hypothesis about the negative pulse of the fluidic oscillator (Fig. 15). The negative pulse, pulls in the liquid as described in the lubrication effect and this effect can be seen herein with a substantial reduction in bubble size and a large difference in bubble size observed for lower viscosity liquids with FO implementation and staging effect. The higher viscosity liquids tend to have a smaller difference observed.

**Fig. 16:**
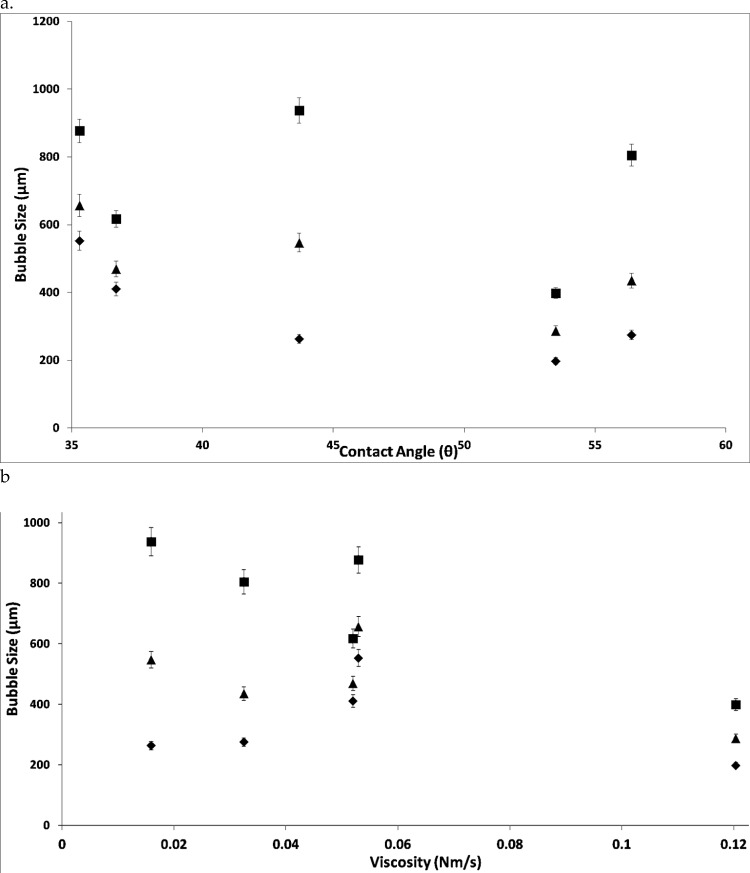
Contact angle vs bubble sizes [left (**a**)] and viscosity vs bubble size [right (**b**)] for various ILs at the three conditions—steady flow, —fluidic oscillator, and —steady flow post-FO

This relationship between the wettability of the liquid for the surface used to engender the bubbles is elucidated when the bubble size is plotted against wettability. This is observed in Fig. 16 where there is no discernible effect seen on the system. This is due to the larger effect observed when viscosity of the system is plotted.

[C_2_mim][DCA] is an example about the difference in the size reduction (maximum pulse transfer and therefore maximum size reduction observed) and the viscosity. The differences in size observed for the other ILs reduces due to the increase in viscosity. The liquid back flow is further increased due to the closed system approximated here. The increased wetting of the liquid results in an even distribution of bubble size due to the even flow distribution as for any case of membrane. Equal responsiveness can only be an assumption. Effectively, there is no such thing as an equally responsive membrane. Therefore, when there is an increased pressure head (due to a highly viscous liquid for example), the wettability increase offered by the negative cycle of the oscillator contributes to the decrease in bubble size and concomitant increase in throughput/effective utilisation of the membrane.

As discussed in Eq. 14, ${N}_{\mathrm{av}}$ is the average bubble size due to the number of bubbles.

The steady-flow size for each liquid as the divisor results in

17 $$\frac{{N}_{\mathrm{avSF}}}{{N}_{\mathrm{avx}}},$$

where *x* takes the cases of *x* = steady flow, *x* = fluidic oscillator, and *x* = steady-flow post-fluidic oscillator application.

Table 2 summarises the bubble sizes for the various conditions achieved coupled with throughput, viscosity, and contact angle.

**Fig. 17:**
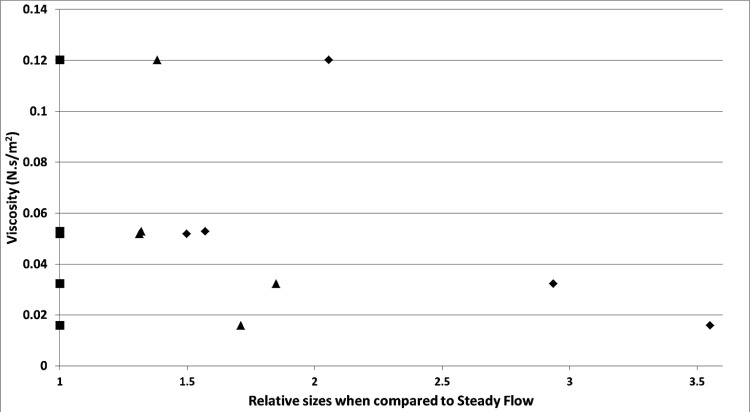
Relative bubble sizes for various ionic liquids at different conditions and size reduction observed. Greater is the distance, higher is the size reduction, and therefore smaller is the bubble size compared to steady flow. The bubble size difference is related to the viscosity of the liquid. Lesser the viscosity of the liquid, greater is the difference and by extension, greater is the effect of staging on the system, unless there are other factors at play, such as for [C_2_mim][EtSO_4_] discussed previously. —fluidic oscillator, —steady flow, and —steady flow post-FO

Relative bubble sizes are taken when the steady-flow bubble size is taken to be the divisor and this helps understand the effect of staging (Fig. 17). In this study, we see the effect of viscosity on the effect of staging and the relative size difference (effectiveness of fluidic oscillator application) on the size reduction observed in the ionic liquids.

**Fig. 18:**
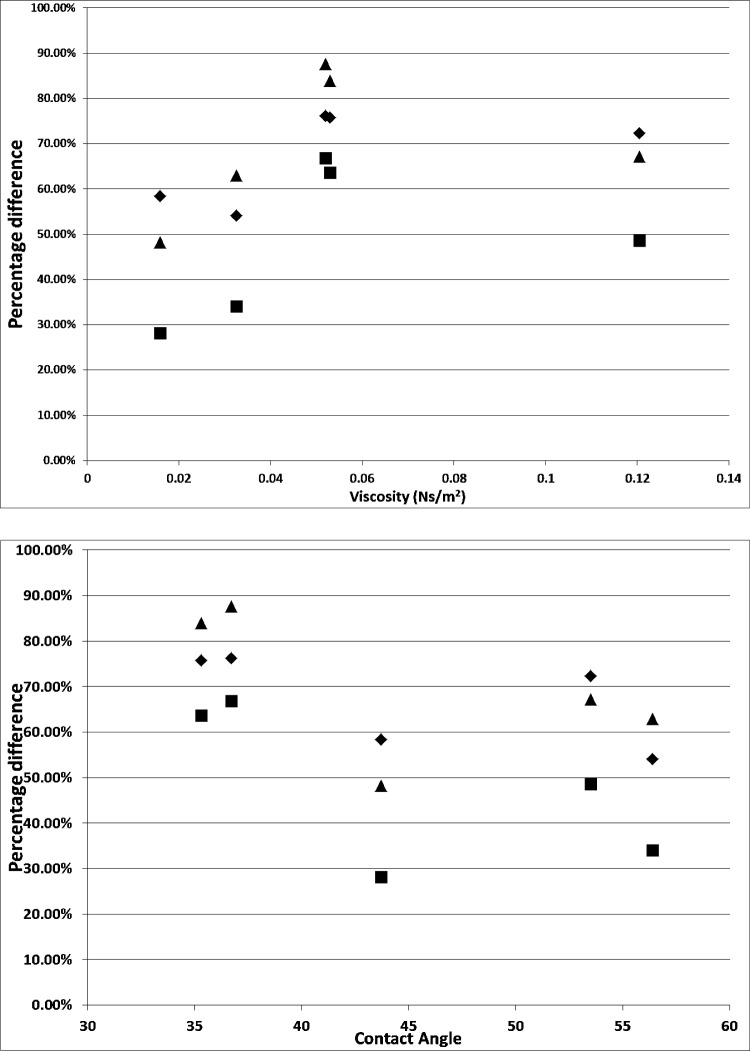
Percentage difference for bubble sizes at various conditions in various liquids versus their viscosities and contact angle. —$\frac{\text{steady flow post}-\mathrm{FO}}{\text{steady flow}}$, —$\frac{\mathrm{FO}}{\text{steady flow}}$, and —$\frac{\mathrm{FO}}{\text{steady flow post}-\mathrm{FO}}$

The percentage difference between the size of the bubble and the various conditions results in Fig. 18

Steady flow post-FO/steady flow is roughly between 50 and 80% of the steady flow bubble size. This is the staging effect taking place and this demonstrates how the viscosity and wettability play an integral part in the sizing.

When FO/steady-flow ratio is plotted, it provides a strong relationship between wetting and bubble size with the viscosity playing a supplementary role in this, thereby resulting in reduced bubble size. This is an important conclusion as this is not apparent but agrees well with the hypothesis for the lubrication effect and previous studies [53].

The FO/steady flow post-FO demonstrates the ‘character’ percentage observed for the morphed system and how much the steady flow post-FO is compared to the FO. Here, the lower viscosity means that there will be a lower value. Higher the viscosity, higher is this value, which means that there is a greater staging observed.

## Conclusions

Bubble size depends on a variety of factors as determined by Taylor et al*.* [55].

Application of the fluidic oscillator reduces the bubble size for all RTILs with viscous forces and wetting forces dominating. The reduction in size is hugely applicable for mass transfer and heat transfer operations.

Application of the fluidic oscillator leaves a ‘memory imprint’ on the membrane and is able to influence membrane performance post-removal. This hypothesis of staging is effective and positively indicated, i.e., negative pulse of the fluidic oscillator results in bubble size reduction based on wettability and viscosity of the liquid, and is additionally responsible for the ‘memory’ of the membrane.

Bubble size distribution for the bubbles for steady-flow post-fluidic oscillation morphs between steady flow and oscillatory flow conditions. This combines the two conditions and results in a mixture of oscillatory and steady-flow bubble size distributions.

Bubble size reduction of the ‘memory imprinted’ membrane is typically 50% of the conventional steady-flow system and so is the observable bubble throughput which is 50–100% higher than the average except in case of [C_2_mim][NTf_2_] which has a threefold increase in throughput.

## Supplementary Information

Below is the link to the electronic supplementary material.

Supplementary file1 (DOCX 359 kb)

## Data Availability

All the data produced from these ionic liquid experiments is embedded in the graphics.
