# Supplementary material for: The effect of staging of fluidic oscillation on microbubble generation in viscous liquids
Source: Eur Phys J Spec Top. 2025 Sep 24;235(1):121–44. doi: 10.1140/epjs/s11734-025-01927-y (PMC13086715; doi:10.1140/epjs/s11734-025-01927-y)

**Supplementary Information**

Bubble size distributions for the 5 ILs
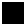
- Steady flow,
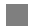
 - Fluidic oscillator, and
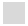
- Steady flow post fluidic oscillator implementation.

**C2mimNTF2**


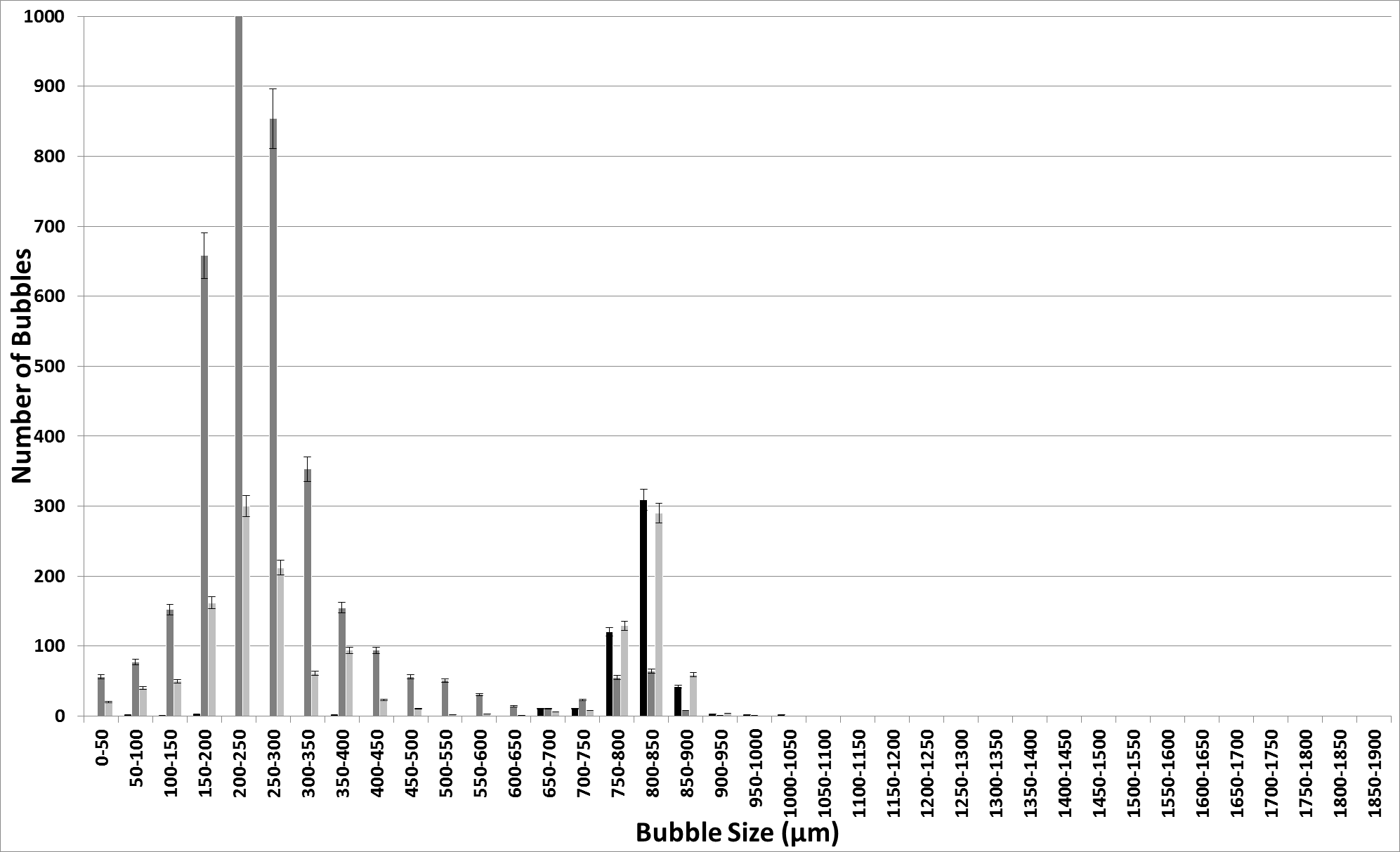


**C4mimNTF2**
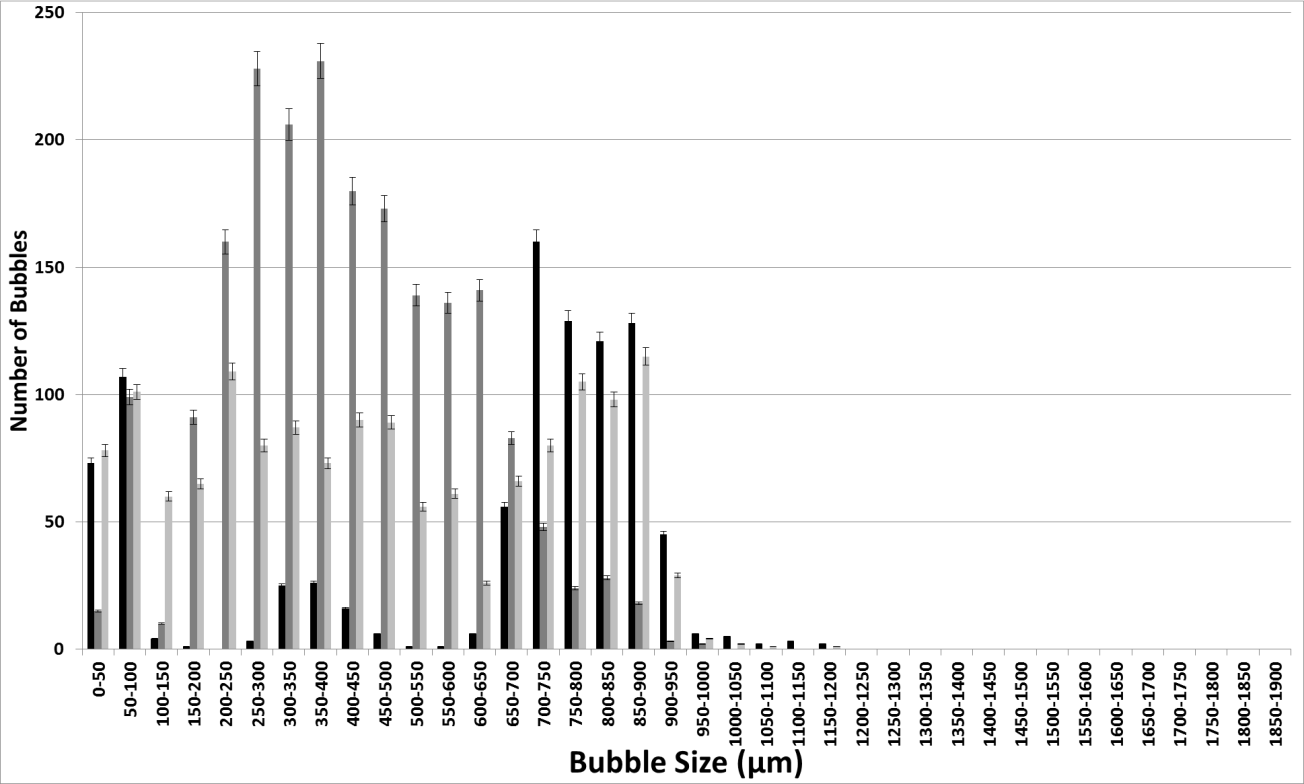


**C4mim TFA**


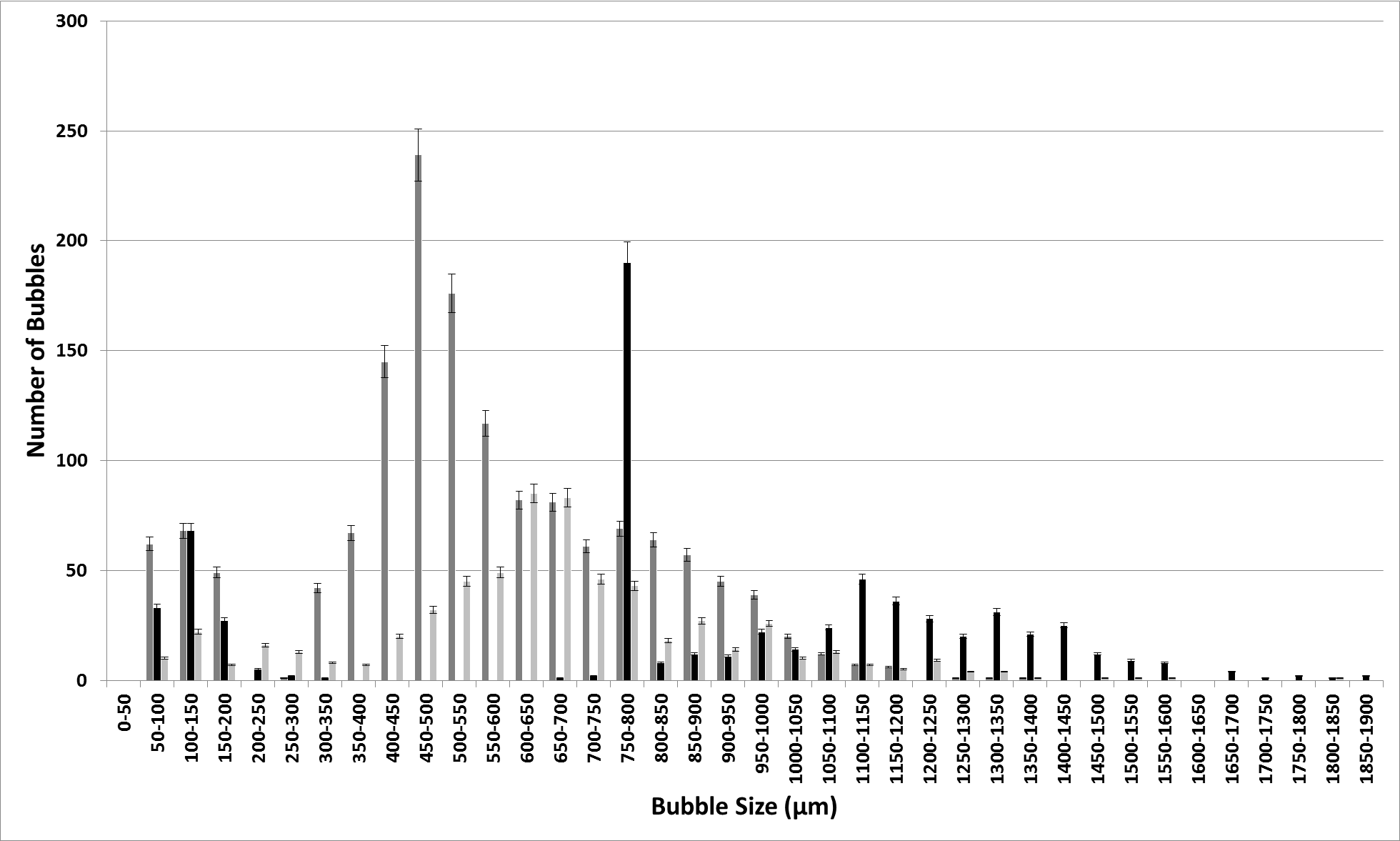


**C2mimETSO4**


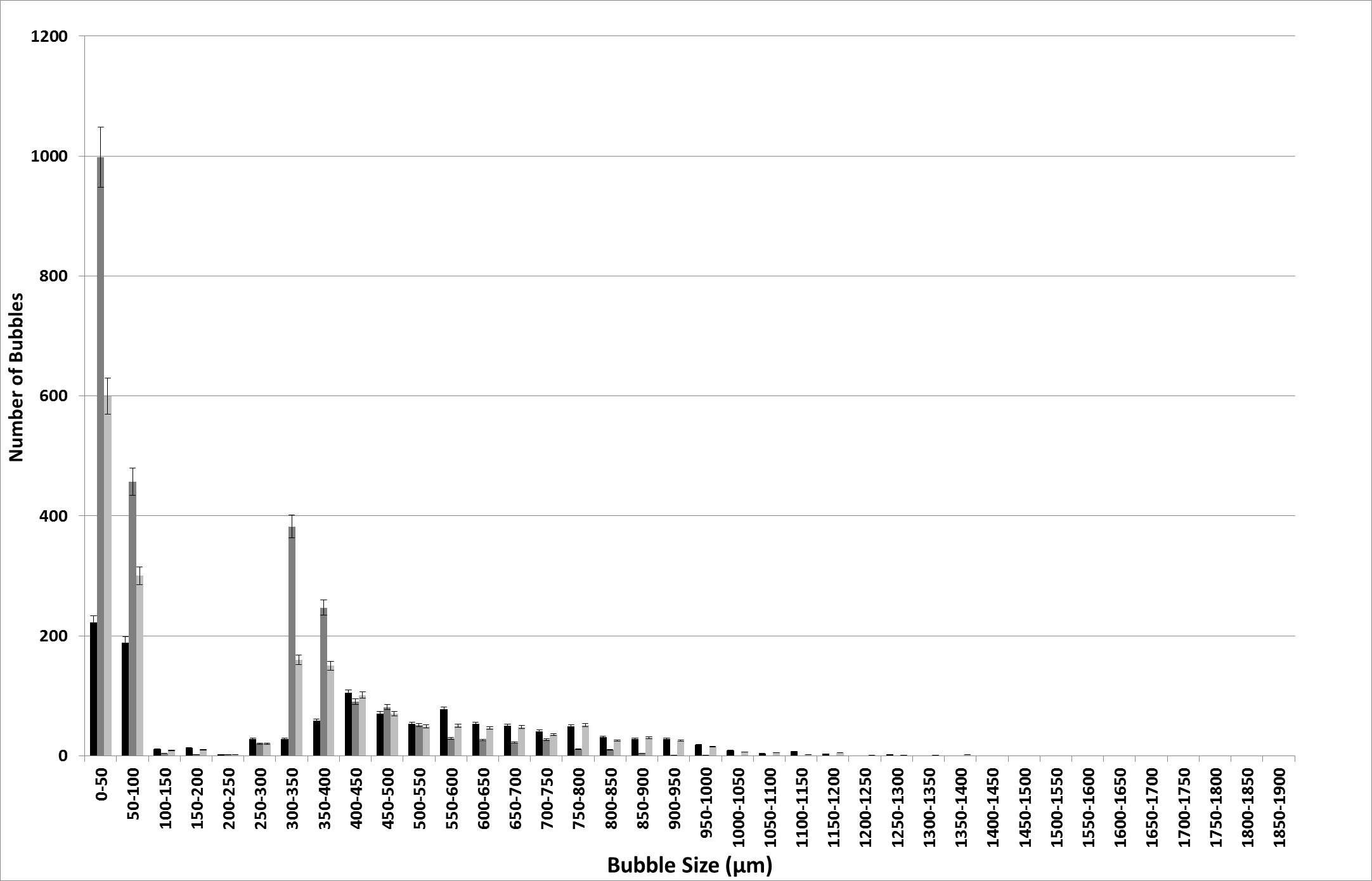


**C2mimDCA**


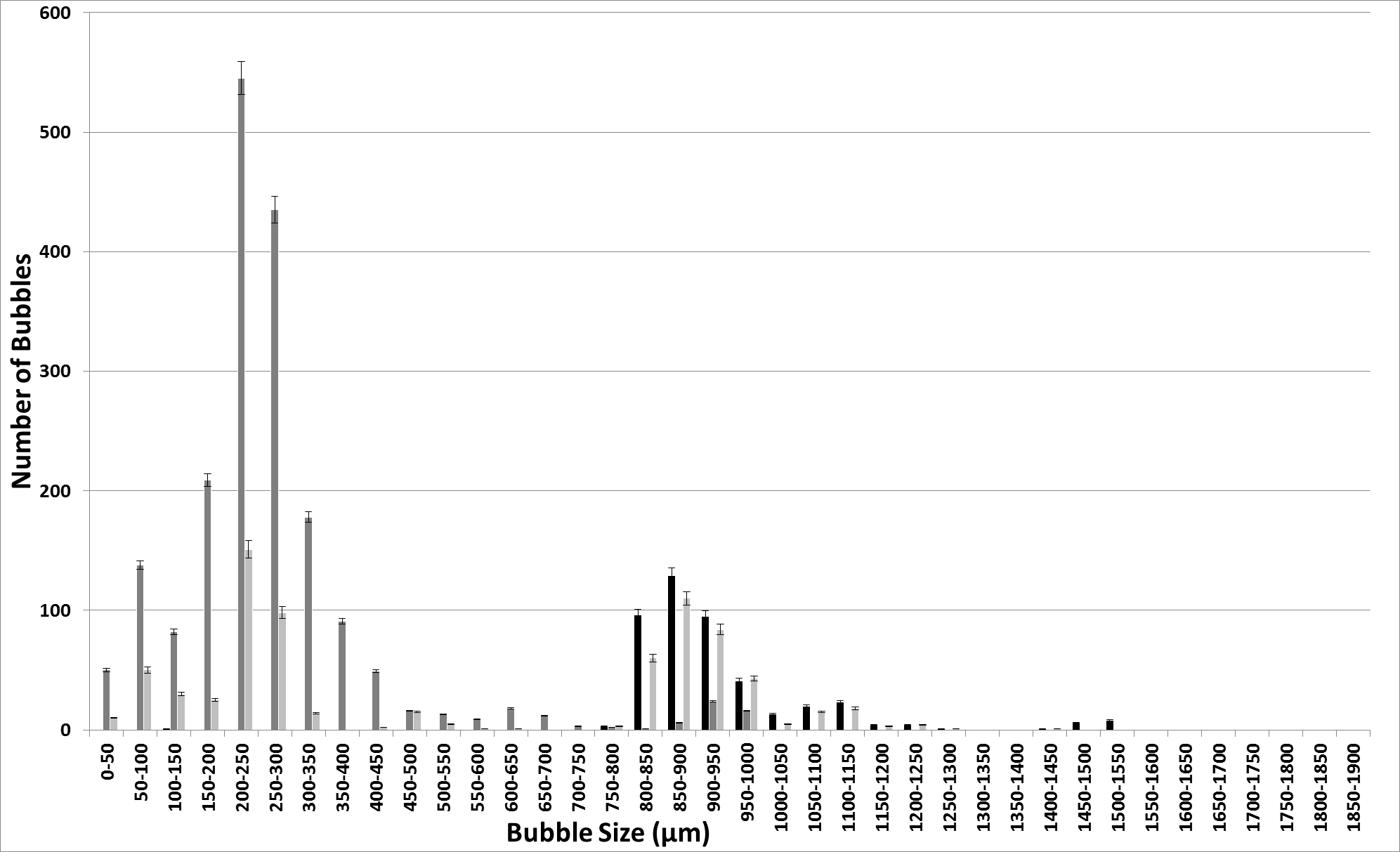

Supplement: Supplementary file 1 — Supplementary file1 (DOCX 359 kb) [file 11734_2025_1927_MOESM1_ESM.docx]
